# Obatoclax, a BH3 Mimetic, Enhances Cisplatin-Induced Apoptosis and Decreases the Clonogenicity of Muscle Invasive Bladder Cancer Cells via Mechanisms That Involve the Inhibition of Pro-Survival Molecules as Well as Cell Cycle Regulators

**DOI:** 10.3390/ijms20061285

**Published:** 2019-03-14

**Authors:** Thomas M. Steele, George C. Talbott, Anhao Sam, Clifford G. Tepper, Paramita M. Ghosh, Ruth L. Vinall

**Affiliations:** 1 Department of Pharmaceutical & Biomedical Sciences, California Northstate University College of Pharmacy (CNUCOP), Elk Grove, CA 95757, USA; THOMAS.STEELE@va.gov or Thomas.steele@cnsu.edu (T.M.S.); GTalbott@cnsu.edu (G.C.T.); Anhao.Sam6546@cnsu.edu (A.S.); 2 VA Northern California Health Care System (VANCHCS), Sacramento, CA 95655, USA; paghosh@ucdavis.edu or Paramita.Ghosh01@va.gov; 3 Department of Urologic Surgery, University of California, Davis, School of Medicine, Sacramento, CA 95817, USA; 4 Department of Biochemistry and Molecular Medicine, University of California, Davis, School of Medicine, Sacramento, CA 95817, USA; cgtepper@ucdavis.edu

**Keywords:** bladder cancer, BH3 mimetics, Obatoclax, cisplatin, apoptosis

## Abstract

Several studies by our group and others have determined that expression levels of Bcl-2 and/or Bcl-xL, pro-survival molecules which are associated with chemoresistance, are elevated in patients with muscle invasive bladder cancer (MI-BC). The goal of this study was to determine whether combining Obatoclax, a BH3 mimetic which inhibits pro-survival Bcl-2 family members, can improve responses to cisplatin chemotherapy, the standard of care treatment for MI-BC. Three MI-BC cell lines (T24, TCCSuP, 5637) were treated with Obatoclax alone or in combination with cisplatin and/or pre-miR-34a, a molecule which we have previously shown to inhibit MI-BC cell proliferation via decreasing Cdk6 expression. Proliferation, clonogenic, and apoptosis assays confirmed that Obatoclax can decrease cell proliferation and promote apoptosis in a dose-dependent manner. Combination treatment experiments identified Obatoclax + cisplatin as the most effective treatment. Immunoprecipitation and Western analyses indicate that, in addition to being able to inhibit Bcl-2 and Bcl-xL, Obatoclax can also decrease cyclin D1 and Cdk4/6 expression levels. This has not previously been reported. The combined data demonstrate that Obatoclax can inhibit cell proliferation, promote apoptosis, and significantly enhance the effectiveness of cisplatin in MI-BC cells via mechanisms that likely involve the inhibition of both pro-survival molecules and cell cycle regulators.

## 1. Introduction

The current 5-year survival rate for patients with muscle invasive bladder cancer (MI-BC) is estimated to be approximately 50% [1]. Several clinical studies support neoadjuvant chemotherapy (NAC), primarily in the form of platinum-based DNA damaging agents such as cisplatin, as being the standard of care therapy for MI-BC patients [2,3]. While NAC does improve overall survival rates, only approximately half of all patients benefit from this treatment option [4]. Chemoresistance to therapy is a major contributing factor to this low response rate.

There are multiple mechanisms that can lead to chemoresistance and thereby the non-response or diminished response of a patient to chemotherapy [5,6]. These include increased DNA repair, formation of trapping agents (e.g., some tumor cells produce glutathione which can trap and thereby inhibit activity of certain anti-cancer drugs), alterations in target molecules, decreased activation of prodrugs, inactivation of anticancer drugs, decreased drug accumulation (e.g., due to increased expression of P-glycoprotein (MDR1)), and increased expression of pro-survival molecules. Recent genomic and RNA sequencing studies of bladder cancer cell lines and/or patient tumors have revealed that alterations in genes associated with DNA repair, cell cycle, and apoptosis occur with the highest frequency in bladder cancer [7,8]. Affected genes include *ATM*, *ERCC1*, *TP53*, *MDM2*, *CDKN2A*, *RB1*, *CDKN1A*, *PTEN*, *CCNE1*, *FBXW7*, *CDKN1B*, *CCND1/2/3*, *CDK4/6*, *TSC1*, *FGFR3*, *PIK3CA*, *PPARG*, *E2F3*, *EGFR*, *ERBB2*, *YAP1*, *MYC*, *ZNF703*, *MYCL1*, and *BCL2L1*. Subsequent studies of some of these genes have confirmed that their altered activity and/or expression level can contribute to chemoresistance in MI-BC cells. For example, overexpression of *BCL2L1* and its protein product, Bcl-xL, has been shown to occur in MI-BC tumors and cell lines and cause resistance to cisplatin and other chemotherapies which are used to treat MI-BC [7,9,10]. Alterations in these genes may affect intrinsic and/or de novo (also referred to as acquired) chemoresistance, thereby impacting initial responses to first line chemotherapy as well as contributing to subsequent treatment failure [11,12].

The development and use of drugs which target the pro-survival members of the Bcl-2 family such as Bcl-2 and Bcl-xL is becoming an increasingly common strategy to combat intrinsic resistance to first line chemotherapy in multiple cancer types [13,14,15,16,17]. These drugs have also shown success as single agents to treat cancers which are driven by dysregulation of apoptosis [18]. Several approaches to inhibit the pro-survival members of the Bcl-2 family have been employed, including the development of anti-sense drugs and synthetic peptides [19,20,21]; however, BH3 mimetics appear to be the most successful of these [14]. BH3 mimetics prevent the binding of pro-survival members of the Bcl-2 family to pro-apoptotic members, thereby allowing for the dimerization of the pro-apoptotic members and activation of the intrinsic pathway of apoptosis. There are currently six BH3 mimetics in clinical development, and one of these, Venitoclax, has FDA approval for the treatment of chronic lymphocytic leukemia (CLL) [13,14,18]. All of these drugs have shown success in both hematological cancers as well as solid cancers. As would be expected, BH3 mimetics are most effective in patients whose tumors overexpress pro-survival members of the Bcl-2 family. In CLL patients, high levels of Bcl-2 expression are driven by dysregulation of miR-15/16 expression as well as chromosomal rearrangements [22]. Other reasons for the overexpression of pro-survival Bcl-2 members include gene amplification (e.g., diffuse large B-cell lymphomas), chromosomal translocation (e.g., Hodgkin’s lymphoma), and alterations in promoter methylation (e.g., bladder cancer) [23,24,25]. The successful usage of BH3 mimetics to reduce chemoresistance in multiple cancer types, along with the knowledge that Bcl-2 and/or Bcl-xL are overexpressed in many MI-BC patients, indicate that the concurrent treatment of BH3 mimetics with cisplatin could improve MI-BC patients’ response rate to NAC.

Another reason we used a BH3 mimetic in our current study was to determine whether it could improve responses to treatment with pre-miR-34a. We previously demonstrated that pre-miR-34a can mediate a dramatic decrease in the clonogenicity of MI-BC cell lines via inhibition of Cdk6, a cell cycle regulator; however, it also caused increased Bcl-2 expression and thereby decreased levels of apoptosis [26]. We hypothesized that treatment with a BH3 mimetic may abrogate this negative effect. miR-34a is a downstream effector of p53 which can regulate the cell cycle, senescence, and apoptosis [27], and its decreased expression can contribute to carcinogenesis and chemoresistance [26,28]. Decreased expression of miR-34a can occur as a result of p53 mutation and/or *miR-34a* gene promoter methylation (both of which are known to occur in MI-BC cells [26,29]) and restoring miR-34a levels has been shown to reduce the impact of the loss of p53 and/or miR-34a function in several cancer types [30]. It is noteworthy that our finding that treatment with pre-miR-34a can inhibit MI-BC clonogenicity has been validated by several recent studies [31,32,33]. This, along with the knowledge that miR-34a expression can be dysregulated in MI-BC cells, indicates that increasing miR-34a expression could be a viable therapeutic strategy. The development and usage of miRNAs as therapeutics and biomarkers is becoming increasing common in the treatment of cancer [34,35]. Major advantages of using miRNAs include the fact that they can simultaneously target multiple molecules and are extremely stable in body fluids. While there are challenges associated with use of miRNA-based therapies in patients, including difficulties in the development of effective delivery methods and unanticipated off target effects, results from on-going studies remain promising [36,37].

The goals of the current study were to determine whether using Obatoclax, a BH3 mimetic, in combination with cisplatin and/or pre-miR-34a, can further inhibit the cell proliferation and clonogenicity of MI-BC cell lines as well as promoting apoptosis, and to identify the potential molecular mechanisms involved.

## 2. Results

### 2.1. Treatment of Muscle Invasive Bladder Cancer Cell Lines with Obatoclax Inhibits Cell Proliferation and Clonogenicity and Promotes Apoptosis in a Dose-Dependent Manner

**Figure 1:**
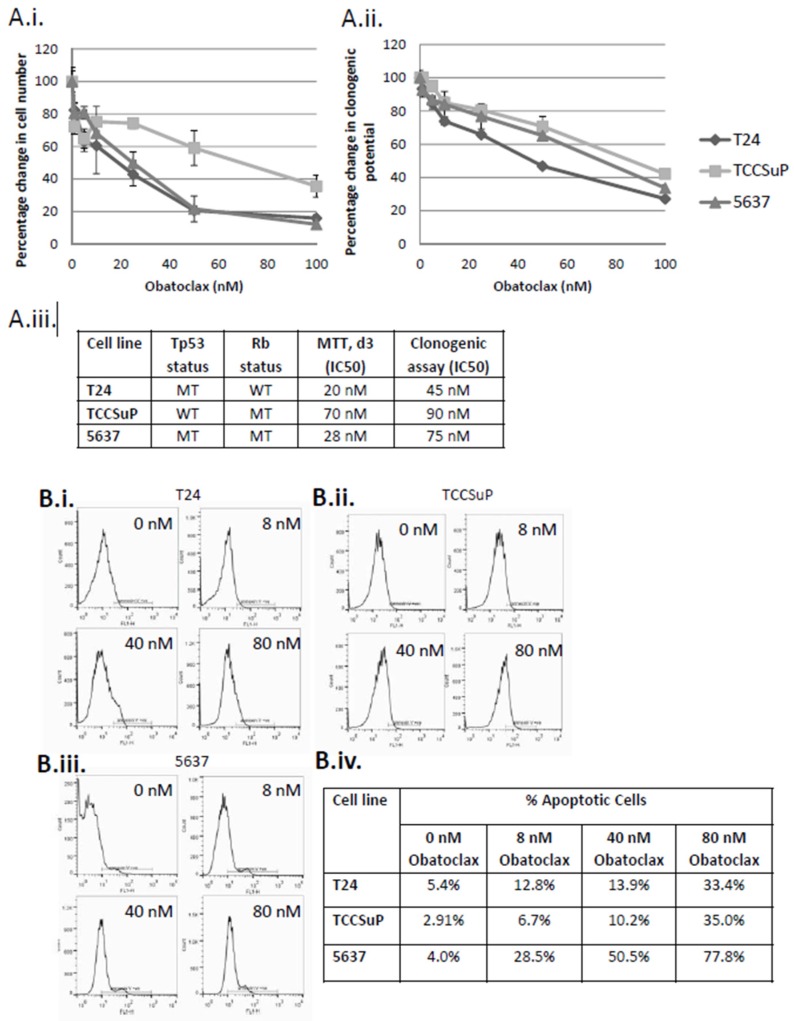
Obatoclax inhibits proliferation and clonogenic potential and promotes apoptosis of muscle invasive bladder cancer cells in a dose-dependent manner. Treatment of T24, TCCSuP, and 5637, muscle invasive bladder cancer (MI-BC) cells with Obatoclax mediated a dose-dependent decrease in cell proliferation (**A.i**) and clonogenic potential (**A.ii**). T24 cells, which harbor a *Tp53* mutation, were the most sensitive to Obatoclax treatment (MTT assay IC_50_ = 20 nM, clonogenic assay IC_50_ = 45 nM), followed by 5637 cells, which harbor a *Tp53* mutation and an *Rb* mutation (MTT assay IC_50_ = 28 nM, clonogenic assay IC_50_ = 75 nM), then TCCSuP, which harbor an *Rb* mutation (MTT assay IC_50_ = 70 nM, clonogenic assay IC_50_ = 90 nM) (**A.iii**). Treatment with Obatoclax also mediated a dose-dependent increase in levels of apoptosis (**B.i**–**iv**). The highest levels of apoptosis were observed in 5637 cells, followed by TCCSuP cells, then T24 cells (treatment with the highest dose of Obatoclax (80 nM) resulted in 77.8%, 35.0%, and 33.4% apoptosis, respectively). These dose-dependent responses to Obatoclax indicate that Obatoclax is acting in a specific manner to inhibit cell proliferation and clonogenicity and to promote apoptosis. It is noteworthy that all of the doses used in this study fall within the physiologically acceptable dose ranges for Obatoclax which have been established through clinical trials [38]. **B.i**–**iii**.; *x*-axis = FL1-H (FITC), *y*-axis = cell count, line bar = annexin V positive cells.

Three cells lines that were originally derived from patients with muscle invasive bladder cancer (MI-BC) were used for these studies; T24, TCCSuP, and 5637. All cell lines showed a dose-dependent response to Obatoclax treatment in both cell proliferation and clonogenic assays (Figure 1A). T24 cells, which harbor a *Tp53* mutation, were the most sensitive to Obatoclax treatment (MTT assay IC_50_ = 20 nM, clonogenic assay IC_50_ = 45 nM), followed by 5637 cells, which harbor a *Tp53* mutation and an *Rb* mutation (MTT assay IC_50_ = 28 nM, clonogenic assay IC_50_ = 75 nM), then TCCSuP, which harbors an *Rb* mutation (MTT assay IC_50_ = 70 nM, clonogenic assay IC_50_ = 90 nM). Annexin V flow cytometry studies demonstrated that Obatoclax also promoted apoptosis in a dose-dependent manner in all 3 cells lines (Figure 1B). The highest levels of apoptosis were observed in 5637 cells, followed by TCCSuP cells, then T24 cells (treatment with the highest dose of Obatoclax (80 nM) resulted in 77.8%, 35.0%, and 33.4% apoptosis, respectively). The dose-dependent responses that are observed indicate that Obatoclax is acting in a specific manner to inhibit cell proliferation and clonogenicity and to promote apoptosis. It is noteworthy that the observed effects all occur within the physiologically acceptable dose ranges of Obatoclax which have been established through clinical trials (Phase I clinical studies conducted on various cancer types showed that the peak plasma concentration (*C*_max_) of Obatoclax ranged from 7 to 39 nM following 24-h infusions and between 34 to 375 nM following 3-h infusions [38]). IC_30_ concentrations of Obatoclax for T24, TCCSuP, and 5637 cells were used for subsequent combination studies to allow us to more easily observe any additive effects (IC_30_ doses used for clonogenic assay-based combination study experiments: 25, 60, and 40 nM, respectively; IC_30_ doses used for Western and apoptosis assay-based combination experiments: 8, 40, and 12 nM, respectively (these IC_30_ doses were calculated using the cell proliferation dose response curves)). Note that IC_30_ values derived from MTT dose curve assays were used for Western and apoptosis assay experiments because the time period between treatment and analysis is comparable (MTT dose curve assay: 1, 3, 5 days; Western and apoptosis assay: 3 days). Clonogenic assays are analyzed 10 days post-treatment. IC_30_ values derived from clonogenic dose curve assays were used for the clonogenic assay-based combination study experiments.

### 2.2. Obatoclax Can Inhibit the Binding of Bcl-2 and Bcl-xL to Bak, and Decreases Expression Levels of Bcl-2 and Bcl-xL

**Figure 2:**
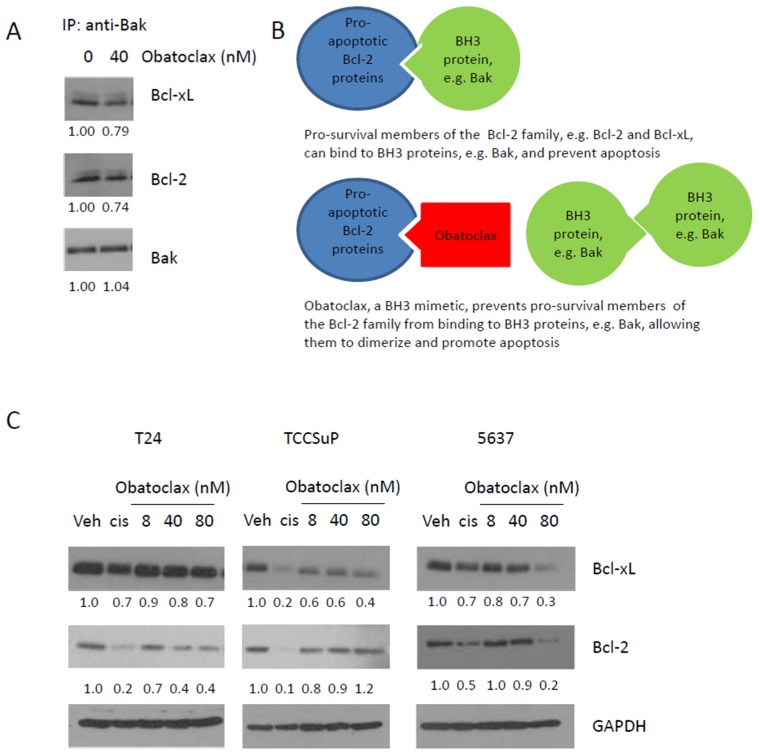
Obatoclax, a BH3 mimetic, is able to inhibit the binding of Bcl-2 and Bcl-xL to Bak and reduces Bcl-2 and Bcl-xL expression levels in muscle invasive bladder cancer cells. Immunoprecipitation analyses demonstrated that the treatment of 5637 cells with 40 nM Obatoclax caused a ~25% decrease in the binding of Bcl-2 and Bcl-xL to Bak (**A**); this finding aligns with the known mechanism of action of Obatoclax (**B**). Obatoclax also mediated reduced expression levels of Bcl-2 and Bcl-xL (**C**). A dose-dependent reduction in Bcl-xL was observed in T24, TCCSuP, and 5637 cells. A dose-dependent reduction in Bcl-2 was observed in T24 and 5637 cells, but not TCCSuP cells. Treatment with cisplatin (5 μM) also resulted in decreased Bcl-2 and Bcl-xL expression (IP = immunoprecipitation, Veh = vehicle control, Cis = cisplatin).

Obatoclax is a BH3 mimetic. Immunoprecipitation experiments using 5637 cells confirmed that Obatoclax can inhibit the binding of Bcl-2 and Bcl-xL to Bak (a BH3 protein); treatment with 40 nM Obatoclax mediated a ~25% decrease in binding of Bcl-2 and Bcl-xL to Bak (Figure 2A). This finding aligns with the known mechanism of action of Obatoclax (Figure 2B). Western blot analyses determined that treatment with Obatoclax causes a dose-dependent decrease in Bcl-xL expression levels in T24, TCCSuP, and 5637 cells (minimal decreases in Bcl-xL expression levels were observed in T24 and TCCSuP cells, a more dramatic decrease was observed in 5637 cells). Obatoclax also causes a dose-dependent decrease in Bcl-2 expression levels in T24 and 5637 cells (Figure 2C). Obatoclax caused a minimal decrease in the expression of Bcl-2 in TCCSuP but did not appear to act in a dose-dependent manner (Figure 2C). Treatment with cisplatin (5 uM) also resulted in decreased Bcl-2 and Bcl-xL expression.

### 2.3. Combining Obatoclax with Cisplatin, the Standard of Care Treatment for Muscle Invasive Bladder Cancer, Can Further Decrease Cell Clonogenicity and Increase Apoptosis

**Figure 3:**
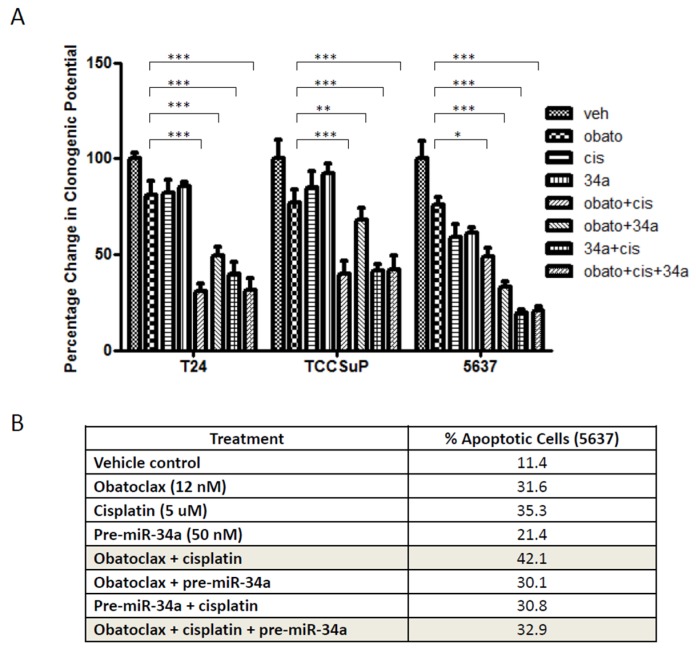
Combining Obatoclax with cisplatin further reduces clonogenic potential and further increases apoptosis. The inclusion of pre-miR-34a in combination treatments can abrogate pro-apoptotic effects. Treatment with Obatoclax as a single agent caused a reduction of clonogenicity in all 3 MI-BC cell lines (*p* < 0.001 for all 3 cell lines) (T24, TCCSuP, and 5637 were treated with IC_30_ value concentations of Obatoclax; 25, 60, and 40 nM, respectively) (**A**); Further reductions were observed when Obatoclax was combined with cisplatin (5 μM), the standard of care treatment for MI-BC (~2.5-fold (*p* < 0.001), ~2.0-fold (*p* < 0.001), and ~1.5-fold (*p* < 0.05) reductions were observed in T24, TCCSuP, and 5637, respectively). Combining Obatoclax with pre-miR-34 treatment also caused further reductions in clonogenicity compared to treatment with Obatoclax or pre-miR-34a as single agents (~1.7-fold (*p* < 0.001), ~1.2-fold (*p* < 0.01), and ~2.0-fold (*p* < 0.001) reduction, respectively). In T24 and TCCSuP, the Obatoclax + cisplatin outperformed the Obatoclax + pre-miR-34a treatment (~2.0-fold (*p* < 0.05), ~1.2-fold (*p* < 0.05) reduction, respectively), but the reverse was true in 5637 cells (~2-fold (*p* < 0.001) reduction). Combining cisplatin with pre-miR-34a, or combining all 3 treatments (Obatoclax + cisplatin + pre-miR-34a) did not result in any further reduction in clonogenicity compared to Obatoclax + cisplatin. Apoptosis assays conducted using 5637 cells demonstrated that the Obatoclax + cisplatin outperformed any of the other treatments (**B**). It is noteworthy that combining pre-miR-34a with Obatoclax + cisplatin caused a dramatic decrease in apoptosis compared to treatment with Obatoclax + cisplatin (32.9% compared to 42.1%, respectively). (Veh = vehicle control, Cis = cisplatin, 34a = pre-miR-34a, Obato = Obatoclax). (* = *p* < 0.05, ** = *p* < 0.01, *** = *p* < 0.001)

Treatment of T24, TCCSuP, and 5637 cells with Obatoclax (IC_30_ value concentrations calculated from clonogenic assay dose curve experiments: 25, 60, and 40 nM, respectively) mediated a ~20–25% decrease in clonogenicity, while treatment with cisplatin (5 μM) mediated a ~20% decrease in clonogenicity in T24 and TCCSuP cells and a 40% decrease in clonogenecity in 5637 cells (Figure 3A). Combining Obatoclax and cisplatin treatment mediated a further reduction in clonogenicity; the combination treatment mediated a ~70% decrease in T24 cells, a ~60% decrease in TCCSuP cells, and a 50% decrease in 5637 cells (Figure 3A). Annexin V analyses demonstrated combining Obatoclax (IC_30_ value concentrations calculated from MTT dose curve experiments: 8, 40, and 12 nM for T24, TCCSuP, and 5637, respectively) and cisplatin treatment also mediated a further increase in apoptosis in 5637 cells compared to treatment with single agents: Obatoclax alone = 31.6% apoptosis, cisplatin alone = 35.3% apoptosis, Obatoclax + cisplatin = 42.1% apoptosis (Figure 3B).

### 2.4. Combining pre-miR-34a with Obatoclax and/or Cisplatin Can Decrease Cell Clonogenicity but Also Decreases Levels of Apoptosis

We have previously shown that miR-34a can inhibit Cdk6 expression in MI-BC cell lines and thereby decrease cell proliferation [26]. Our goal here was to determine whether increasing miR-34a expression levels could enhance the efficacy of Obatoclax and/or cisplatin. Combining pre-miR-34a treatment with cisplatin or Obatoclax treatment mediated a further decrease in clonogenicity in all 3 cells lines (Figure 3A). While pre-miR-34a did improve Obatoclax and cisplatin efficacy with regards to their impact on clonogenicity, it was not able to increase levels of apoptosis when used in combination with these drugs; in fact, decreased levels of apoptosis were observed, as combining pre-miR-34a with Obatoclax or cisplatin treatment caused minor decreases in apoptosis of 5637 cells compared to treatment with Obatoclax or cisplatin as single agents (Obatoclax = 31.6%, cisplatin = 35.3%m, pre-miR-34a + Obatoclax = 30.1%, pre-miR-34a + cisplatin = 30.8%). Combining pre-miR-34a with both cisplatin and Obatoclax caused a dramatic decrease in apoptosis (pre-miR-34a + Obatoclax + cisplatin = 32.9% apoptosis while Obatoclax + cisplatin = 42.1%) (Figure 3B). These data from our apoptosis analyses reduce the enthusiasm for pursuing pre-miR-34a as a potential treatment for MI-BC.

### 2.5. Treatment with Obatoclax and Cisplatin Can Further Reduce Bcl-2, Bcl-xL, and Cyclin D1 Expression Levels, While Treatment with pre-miR-34a Reduces Cdk6 and Cyclin D1 Expression Levels

**Figure 4:**
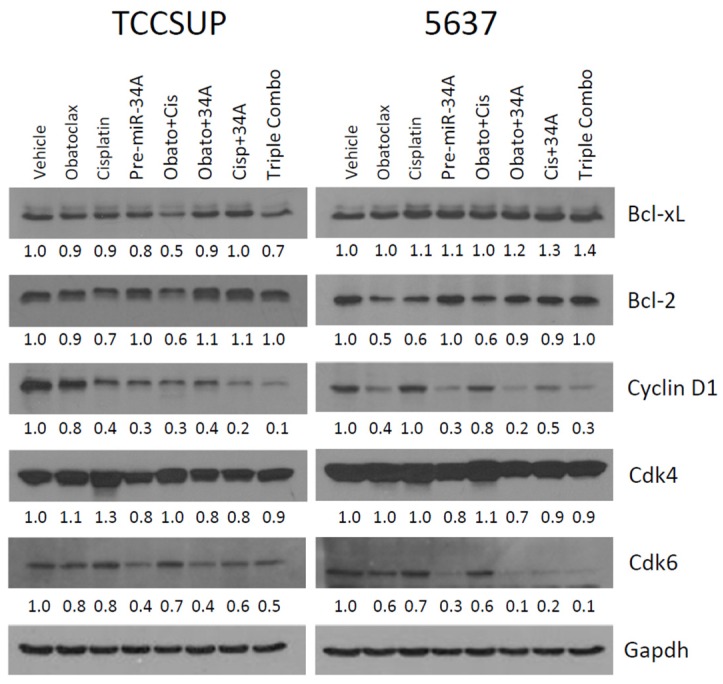
Combining Obatoclax with cisplatin and/or pre-miR-34a can impact expression levels of Bcl-xL and Bcl-2 as well as expression levels of cyclin D1 and cdk4/6. In TCCSuP cells, the Obatoclax + cisplatin combination treatment was the most effective treatment with regards to its ability to decrease the expression levels of Bcl-xl and Bcl-2. In 5637 cells, this combination treatment caused a similar impact on these molecules compared to treatment with Obatoclax as a single agent. Combining pre-miR-34a with Obatoclax and/or cisplatin caused increased levels of Bcl-2 expression both TCCSuP and 5637 cells, and increased levels of Bcl-xL in 5637 cells compared to treatment with Obatoclax and/or cisplatin. Treatment with Obatoclax as a single agent or in combination with cisplatin reduced the expression of cyclin D1 and Cdk6 in both cell lines. Treatment with pre-miR-34a also caused the reduced expression of cyclin D1 and Cdk6; however, it caused increased Bcl-2 expression and negated the effect on Obatoclax on Bcl-2 expression in the combination treatments in both cell lines. Unlike the other treatments, pre-miR-34a was also able to reduce Cdk4 expression levels at the treatment doses used for this experiment (Obatoclax; 25, 60, and 40 nM, respectively, in T24, TCCSuP, and 5637, cisplatin; 5 μM, pre-miR-34a; 50 nm). (Veh = vehicle control, Cis = cisplatin, 34a = pre-miR-34a, Obato = Obatoclax, triple combo = Obatoclax + cisplatin + pre-miR-34a).

Treatment with Obatoclax + cisplatin, the combination treatment which showed highest efficacy in the clonogenic and apoptosis assays (Figure 3A,B), resulted in a further reduction in Bcl-xL, Bcl-2, and cyclin D1 expression levels compared to treatment with Obatoclax and cisplatin as single agents in TCCSuP (Figure 4). In 5637 cells, the Obatoclax + cisplatin combination treatment did not outperform Obatoclax treatment alone in regards to its impact on the expression of these molecules; both Obatoclax alone and Obatoclax + cisplatin reduced Bcl-2 levels by similar amounts, and did not appear to impact Bcl-xL or cyclin D1 expression levels (Figure 4). It is noteworthy that treatment with Obatoclax as a single agent mediated a decreased expression of cyclin D1 and Cdk6 in both cell lines. To our knowledge, the finding that Obatoclax can impact the expression of these molecules in MI-BC cells has not previously been reported.

Our current data confirm our previous finding that pre-miR-34a can mediate the inhibition of Cdk6 expression in MI-BC cells [26] (Figure 4). We now demonstrate that pre-miR-34a can also inhibit the expression of cyclin D1 (Figure 4). The latter observation has not previously been reported. Combining pre-miR-34a with Obatoclax caused a further reduction in Cdk6 and cyclin D1 expression levels in both TCCSuP and 5637 cells (Figure 4), and this observation aligns with the further decrease in clonogenicity that combining pre-miR-34a with Obatoclax mediated (Figure 3A). It is noteworthy that pre-miR-34a caused increased Bcl-2 expression and negated the effect of Obatoclax on Bcl-2 expression in the combination treatments (Figure 4). Increases in Bcl-xL levels were also observed in 5637 cells. These data align with our finding that the concurrent treatment of 5637 cells with pre-miR-34a and Obatoclax and/or cisplatin causes a reduction in levels of apoptosis (Figure 3B).

### 2.6. Confirmation That Obatoclax Can Inhibit Cyclin D1 and Cdk6 Expression Levels, and Can Inhibit Cdk4 Expression Levels

**Figure 5:**
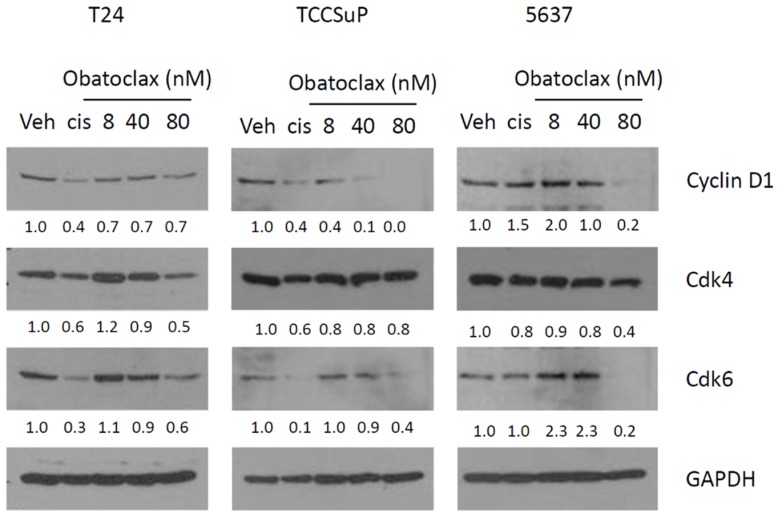
Dose curve experiments confirm that Obatoclax can cause the decreased expression of cyclin D1 and Cdk4/6. Treatment with Obatoclax caused dose-dependent reductions in the expression of cyclin D1 in TCCSuP and 5637 cells. In T24 cells, the highest dose of Obatoclax (80 nM) caused a slight reduction in cyclin D1 expression. A clear dose-dependent reduction in Cdk6 expression levels was observed in all three cell lines, while dose-dependent reductions in Cdk4 expression levels were observed in T24 and 5637 cells. (Veh = vehicle control, Cis = cisplatin).

The ability of Obatoclax to inhibit cyclin D1 and Cdk6 in MI-BC cells has not previously been reported. To confirm that Obatoclax can inhibit the expression of these molecules, we performed dose response experiments (Figure 5). Clear dose-dependent reductions in the expression of cyclin D1 were observed in TCCSuP and 5637 cells. In T24 cells, the highest dose of Obatoclax (80 nM) caused a slight reduction in cyclin D1 expression. A clear dose-dependent reduction in Cdk6 expression levels was observed in T24 and TCCSuP, and the 80 nM inhibited Cdk6 expression in 5637 cells. Decreases in Cdk4 expression were also observed in T24 and 5637 at the 80nM dose of Obatoclax. Again, it is noteworthy that all of the 3 doses used are well within the range of what are considered to be physiological doses for Obatoclax [38]. The combined data provide strong evidence that Obatoclax can inhibit cyclin D1, Cdk4, and Cdk6 at physiologically relevant doses.

## 3. Discussion

Cisplatin is the cornerstone of neoadjuvant chemotherapy (NAC), the standard of care therapy for MI-BC patients [2,3]. To our knowledge, this is the first study to demonstrate that combining Obatoclax with cisplatin can increase levels of MI-BC cell apoptosis and further reduce cell proliferation and clonogenicity compared to treatment with cisplatin as a single agent. The ability of Obatoclax to inhibit cyclin D1, cdk4, and cdk6 in MI-BC cells in addition to its known targets (pro-survival members of the Bcl-2 family) has also not previously been reported.

While our finding that combining Obatoclax with cisplatin increases levels of MI-BC cell apoptosis has not been previously reported, the results are somewhat expected based on our knowledge that the expression of pro-survival members of the Bcl-2 family are elevated in many MI-BC cell lines and tumors and contribute to chemoresistance. Combining inhibitors of pro-survival Bcl-2 family members with cisplatin treatment has been successful in other cancer types which overexpress these molecules. For example, combining small molecule inhibitors with cisplatin treatment can enhance the apoptosis of non-small cell lung cancer, breast cancer, and esophageal cancer cells [39,40,41]. It is noteworthy that another group has shown that combining Obatoclax with paclitaxel treatment increases apoptosis and is able to overcome paclitaxel resistance in urothelial cancer cells (paclitaxel is a second-line treatment for metastatic bladder cancer; it prevents cell division via inhibition of microtubule function) [42]. In their study, they assessed the impact of Obatoclax on Mcl-1 (another pro-survival member of the Bcl-2 family) expression levels and demonstrated that the inhibition of Mcl-1 contributed to Obatoclax-mediated apoptosis. They did not assess Bcl-2 or Bcl-xL expression levels; however, their data are compelling and further support the use of Obatoclax to abrogate pro-survival Bcl-2 family member-mediated intrinsic chemoresistance in bladder cancers.

Our data clearly demonstrate that Obatoclax can inhibit the activity of Bcl-2 and Bcl-xL in MI-BC cells, and can inhibit the expression of these molecules in a dose-dependent manner. We also demonstrate that Obatoclax can inhibit the expression of cyclin D in MI-BC cells. Cyclin D1 is a cell cycle regulator which can drive MI-BC cell proliferation when overexpressed [43]. While our finding that Obatoclax can inhibit cyclin D1 in MI-BC cells was initially unexpected and has not previously been reported, a subsequent review of the literature revealed that two other studies have shown that Obatoclax can inhibit cyclin D1 in colorectal cancer cells [44,45]. In combination with our study, these help validate Obatoclax as being a cell cycle inhibitor in addition to being an inhibitor that promotes apoptosis. Certainly, this dual role increases the attractiveness of Obatoclax as an anti-cancer drug. The colorectal cancer study data indicate that Obatoclax mediates decreased expression of cyclin D1 via accelerating proteasome-mediated cyclin D1 degradation; the inhibition of proteasomal degradation using MG132 abrogated the ability of Obatoclax to cause decreased cyclin D1 expression. Obatoclax triggered the proteasomal degradation of cyclin D1 by causing T286 phosphorylation of cyclin D1 in some but not all colorectal cancer cell lines. The kinase responsible for this phosphorylation was not identified, indicating that an unknown mechanism which promotes proteasomal degradation exists. Clearly, there is much more that needs to be done to fully elucidate the mechanism by which Obatoclax mediates the inhibition of cyclin D1 in cancer cells.

Another goal of the current study was to determine whether Obatoclax could negate pre-miR-34a-mediated increase in Bcl-2. We had previously demonstrated that pre-miR-34a is able to reduce MI-BC proliferation and clonogenicity through the inhibition of Cdk6, a cell cycle regulator, but it is unable to significantly increase levels of apoptosis due to increased Bcl-2 expression [26]. Treatment with Obatoclax was unable to overcome this Bcl-2 response, and in fact combining pre-miR-34a treatment with Obatoclax and/or cisplatin treatment decreased levels of apoptosis. While this negative result was disappointing, the assessment of Cdk6 in our combination study experiments led us to discover that Obatoclax can also inhibit Cdk6. In addition, we found that Obatoclax can inhibit Cdk4, which is also a cell cycle regulator. To our knowledge, the ability of Obatoclax to inhibit Cdk4 and Cdk6 has not previously been described in any cancer type. Inhibiting cancer cell proliferation via targeting Cdk4/6 and other cell cycle regulators is a therapeutic strategy which appears to show much promise in the treatment of several solid tumor types, including bladder cancers [43,46,47,48]. It is noteworthy that the deletion of *CDKN2A* and *CDKN2B*, key regulators of Cdk4/6 expression, occurs in ~50% of bladder cancers, as this indicates that many MI-BC patients may be responsive to Cdk4/6 inhibitors [49].

The ability of Obatoclax to inhibit multiple pro-survival members of the Bcl-2 family, including Bcl-2, Bcl-xL, and Mcl-1, as well as cyclin D1 and Cdk4/6, is important clinically because a comparison of MI-BC patient studies reveals that they differ with regards to which pro-survival Bcl-2 family members are overexpressed [7,50]. The broad spectrum of activity of Obatoclax makes it a viable drug choice regardless of which pro-survival member of the Bcl-2 family is overexpressed in a particular patient. The ability of Obatoclax to target multiple molecules should also allow it to abrogate the development of de novo chemoresistance in addition to intrinsic chemoresistance. While we did not observe increased expression of Bcl-2 or Bcl-xL following treatment with cisplatin as a single agent, others have reported that cisplatin can mediate an enhanced expression of these molecules in MI-BC cells, indicating that the development of de novo chemoresistance to cisplatin may be a concern [51,52,53]. In support of this, prognostic studies have shown that increased expression of pro-survival Bcl-2 family members is observed in some post-chemotherapy MI-BC tumor samples and that this is associated with worse patient outcomes [54,55,56,57]. MI-BC tumor heterogeneity has also been shown to further increase following chemotherapy [50].

In addition to causing cell death by apoptosis, studies in esophageal and ovarian cancer cells have demonstrated that Obatoclax can also induce and/or potentiate autophagy-induced cell death as well as promote necroptosis, a recently described type of cell death that is linked to inflammatory responses [41,58,59,60,61,62]. Several of these studies indicate that the alkalinization of lysosomes may play a role in mediating these effects. The precise mechanisms involved, and whether Obatoclax can mediate autophagy and/or necroptosis as well as apoptosis in MI-BC cells, remain unknown. It will be interesting to see if the inhibition of cyclin D1 and Cdk4/6 as well as the inhibition of pro-survival Bcl-2 family members plays a role in these processes.

In summary, our findings show that combining Obatoclax with cisplatin can increase MI-BC cell apoptosis as well as inhibit cell proliferation and clonogenicity, and that Obatoclax can inhibit cyclin D1 and Cdk4/6 as well as Bcl-2 and Bcl-xL in MI-BC cells; these results are novel and warrant further investigation. Another BH3 mimetic, Venitoclax, has already received FDA approval for the treatment of CLL. This information, combined with the ability of Obatoclax to inhibit multiple molecules that are associated with MI-BC carcinogenesis and resistance to chemotherapy, indicate it could be a viable treatment option for MI-BC and may help improve response rates to NAC. With the current response rate of MI-BC patients to NAC standing at approximately 50%, new treatment options are clearly needed.

## 4. Materials and Methods

### 4.1. Reagents

Antibodies: Bcl-2 (2870S, Cell Signaling, Danvers, MA, USA), Bcl-xL (2762S, Cell Signaling), cyclin D1 (2922S, Cell Signaling), Cdk4 (sc-601, Santa Cruz Biotechnologies, Dallas, TX, USA), Cdk6 (Ab-2 K6.9, Neomarker, Fremont, CA, USA), Gapdh (MAB374, EMD Millipore, Burlington, MA, USA), Bak (AM03, EMD Millipore), IgG (3900S, Cell Signaling), HRP antibodies (W401B (anti-mouse), W402B (anti-rabbit), Promega, Madison, WI, USA). Protein A/G agarose beads were purchased from Cell Signaling. Cisplatin and Obatoclax were purchased from Selleck Chemicals (Houston, TX, USA). Pre-miR-34a and the premiRNA negative control were purchased from Applied Biosystems (Foster City, CA, USA). Pre-miR-34a mimics the product of Dicer cleavage and thereby increases miR-34a activity.

### 4.2. Cell Line and Culture

T24, TCCSuP and 5637 cell lines were obtained from the American Type Culture Collection (ATCC, Manassas, VA, USA). Cells were maintained in RPMI1640 media (Invitrogen/GIBCO, Carlsbad, CA, USA) supplemented with 10% fetal bovine serum (Omega Scientific, Inc., Tarzana, CA, USA), 2 mM l-glutamine, and 100 U/mL penicillin-100 μg/mL streptomycin. Cells were kept at 37 °C in a humidified environment of 5% CO_2_ in air.

### 4.3. Cell Proliferation Assays

Cells were plated in 96-well plates (T24; 1500 cells/well, TCCSuP; 2000 cells/well, 5637; 2000 cells/well). Treatments were initiated after 24 h. MTT (3-[4,5-dimethylthiazol-2-yl]-2,5-diphenyltetrazolium bromide; thiazolyl blue) (Sigma, St Louis, MA, USA) assays were performed to assess cell proliferation as previously described [63]. 

### 4.4. Clonogenic Assay

Cells were plated into 35-mm culture dishes (T24; 2500 cells, TCCSUP; 3500 cells, 5637; 2500 cells) and allowed to attach for 24 h at 37 °C prior to treatment. Following treatment, cells were cultured for 10 days. After 10 days, colonies were fixed in 1.0% crystal violet and 0.5% glacial acetic acid in ethanol. Visible colonies containing approximately 50 or more cells were then counted.

### 4.5. Annexin V Flow Cytometry

The TACS annexin V-FITC kit (R&D Systems, Minneapolis, MN, USA) was used to quantitate apoptosis as per manufacturer’s protocol. A Coulter Epics XL flow cytometer (Beckman Coulter, Indianapolis, IN, USA) was used to perform the flow cytometry analyses. Compensation analyses were performed using FlowJo software (FlowJo, Ashland, OR, USA).

### 4.6. Pre-miR-34 Transfection

Lipofectamine 2000 (Invitrogen, Carlsbad, CA, USA) was used for pre-miR-34a transfections as per manufacturer’s instructions.

### 4.7. Western Blot

Analyses were performed as previously described [63].

### 4.8. Immunoprecipitation

Cells were lysed in immunoprecipitation (IP) buffer supplemented with a protease inhibitor tablet (both from Thermo Fisher, Waltham, MA, USA). One milligram of protein was incubated with 4 ug anti-Bak antibody (AM03, EMD Millipore) overnight at 4 °C followed by incubation with 50 μL of protein A/G agarose beads (Cell Signaling) for 2 h at 4 °C and then washed with IP buffer. Immunoprecipitates were boiled in sample buffer for 5 min.

### 4.9. Statistical Analyses

At least three independent experiments were completed for each analysis described in this article. Data are shown as mean ± standard deviation. Multiple group comparisons were performed by ANOVA followed by the Bonferroni post-test procedure for comparison of means using a commercially available software program (GraphPad Prism, GraphPad Software, La Jolla, CA, USA). *p* < 0.05 was considered statistically significant.

## Abbreviations

Cis	Cisplatin
CLL	Chronic lymphocytic leukemia
*C*_max_	Peak plasma concentration
IC_30_	Thirty percent of maximal inhibitory concentration
IC_50_	Half maximal inhibitory concentration
IP	Immunoprecipitation
MI-BC	Muscle invasive bladder cancer
NAC	Neoadjuvant chemotherapy
Veh	Vehicle
